# Prognostic and clinicopathological significance of tertiary lymphoid structure in non-small cell lung cancer: a systematic review and meta-analysis

**DOI:** 10.1186/s12885-024-12587-x

**Published:** 2024-07-08

**Authors:** Luyuan Ma, Rongyang Li, Xiaomeng Liu, Wenhao Yu, Zhanpeng Tang, Yi Shen, Hui Tian

**Affiliations:** 1 Department of Thoracic Surgery, Qilu Hospital of Shandong University, Jinan, 250012 Shandong China; 2 Department of Pediatrics, Qilu Hospital of Shandong University, Jinan, 250012 Shandong China

**Keywords:** Tertiary lymphoid structures, Non-small cell lung cancer, Prognosis, Systematic review, Meta-analysis

## Abstract

**Background:**

Non-small cell lung cancer (NSCLC) is the primary reason for cancer-related deaths globally. Tertiary lymphoid structure (TLS) is an organized collection of immune cells acquired in non-physiological, non-lymphoid tissues. High expression of TLS in tumor tissues is generally associated with better prognosis. This research aimed to investigate the prognostic and clinicopathological significance of TLS in patients with NSCLC.

**Methods:**

A comprehensive literature search was conducted based on Pubmed, EMBASE, and Cochrane Library databases to identify eligible studies published up to December 8, 2023. The prognostic significance and clinicopathological value of TLS in NSCLC were evaluated by calculating the combined hazard ratios (HRs) and odds ratios (ORs) and their 95% confidence intervals (CIs). Following that, additional analyses, including subgroup analysis and sensitivity analysis, were conducted.

**Results:**

This meta-analysis evaluated the prognostic and clinicopathological significance of TLS in 10 studies involving 1,451 patients with NSCLC. The results revealed that the high levels of TLS were strongly associated with better overall survival (OS) (HR = 0.48, 95% CI: 0.35–0.66, *p* < 0.001), disease-free survival (DFS)/recurrence-free survival (RFS) (HR = 0.37, 95% CI: 0.24–0.54, *p* < 0.001), and disease-specific survival (DSS) (HR = 0.45, 95% CI: 0.30–0.68, *p* < 0.001) in NSCLC patients. In addition, the increased expression of TLS was closely related to the Tumor Node Metastasis (TNM) stage of tumors (OR = 0.71, 95% CI: 0.51-1.00, *p* < 0.05) and neutrophil-lymphocyte ratio (NLR) (OR = 0.33, 95% CI: 0.17–0.62, *p* < 0.001).

**Conclusions:**

The results revealed that highly expressed TLS is closely associated with a better prognosis in NSCLC patients. TLS may serve as a novel biomarker to predict the prognosis of NSCLC patients and guide the clinical treatment decisions.

**Supplementary Information:**

The online version contains supplementary material available at 10.1186/s12885-024-12587-x.

## Introduction

Lung cancer is one of the most prevalent cancers globally, with high death rates in both genders. The majority of lung cancers are attributed to non-small cell lung cancer (NSCLC), causing the most cancer-related deaths and ranking as the second most prevalent cancer globally [1–3]. In recent years, despite great progress in multidisciplinary treatment including surgery, radiotherapy, chemotherapy and immunotherapy, the prognosis of patients with NSCLC remains unsatisfactory. Hence, it is crucial to identify significant prognostic biomarkers for NSCLC to improve the clinical management of patients.

Tertiary lymphoid structure (TLS) is an abnormal lymphoid organ that closely resembles the secondary lymphoid organ (SLO) [4]. Under normal circumstances, TLS does not typically occur in the body, instead, it is found in non-lymphoid tissues where chronic inflammation is present [5]. TLS can develop in different pathophysiological conditions such as autoimmune diseases, infectious diseases, and tumors, leading to various effects that are influenced by the environment [6]. Recently, high levels of TLS have been proven to be linked to improved prognosis in various types of cancer [7, 8]. In patients with breast cancer, elevated levels of TLS is strongly related to a positive outlook for their prognosis [9]. In cases of gastrointestinal tumors, TLS can serve as a valuable prognostic indicator for gastrointestinal cancer and help direct the use of cancer immunotherapy [10]. Although numerous studies have explored the significance of TLS in predicting survival outcomes for NSCLC patients, the prognostic and clinicopathological significance of TLS in NSCLC remains controversial. For example, Brunet et al. found that progression-free survival (PFS) in the group of patients with TLS-positive tumors were not significantly different from patients with TLS-negative tumors [11]. However, some other studies have shown that elevated expression of TLS is closely related to better prognosis of patients with resectable NSCLC [12, 13].

In the present study, we conducted a meta-analysis to investigate the prognostic value of intratumoral TLS in patients with NSCLC, with the aim of providing evidence regarding the potential of TLS as a novel prognostic biomarker for NSCLC.

## Materials and methods

### Protocol and ethics statement

The reports of this systematic review and meta-analysis are in line with the Preferred Reporting Project for Systematic Review and Meta-Analysis (PRISMA) and the Meta-Analysis of Observational Epidemiological Studies (MOOSE) guidelines and statements [14, 15]. This systematic review and meta-analysis protocol has been registered on the PROSPERO website (https://www.crd.york.ac.uk/PROSPERO/) with the registration number CRD42024504484. All data used in this meta-analysis were from published studies, so ethical approval and patient consent were not required for this study.

### Databases and search strategy

Two authors (Luyuan Ma and Rongyang Li) independently searched and assessed the availability of studies in each of the three databases: PubMed, EMBASE and the Cochrane Library, up to December 8th, 2023. Medical subject terms (MeSH) in the search strategy include “Tertiary lymphoid structure” and “Pulmonary Neoplasms” and “Prognosis”, and looked up free terms on PubMed. Various possible combinations of keywords and free words are made through two Boolean operators (“AND “and “OR”). The detailed search strategies for all databases are shown in Supplementary Table 1. In addition, we reviewed references in relevant articles for potential studies. Any disagreement between two reviewers is resolved by inviting other reviewers to discuss it.

### Study selection and criteria

The primary studies included in this meta-analysis satisfied all of the criteria as follows: (I) This research focuses on individuals diagnosed with NSCLC. (II) Expression level of TLS in tumor tissues was clearly detected. (III) There are clear TLS grouping standards, which divide TLS into high/low expression groups for analysis and research. (IV) The relationship between TLS and survival outcomes or clinicopathological characteristics was evaluated in studies. Meanwhile, we excluded non-compliant studies by using the following criteria: (I) Reviews, meta-analyses, case reports, conference abstracts, letters and comments. (II) Animal experiments or basic research. (III) Studies that don’t have enough data to analyze. (IV) Multiple studies utilizing the same set of samples or participants.

### Data extraction and quality assessment

From each of the studies that were included, we extracted the following information: authors, year of publication, country, study design, sample size, treatment, TLS detection methods, TLS location, cut-off criteria of TLS, follow-up time and survival outcomes. In addition, we collected the association of TLS with the age, gender, pathologic staging, smoking, Tumor Node Metastasis (TNM) staging and neutrophil-lymphocyte ratio (NLR) for patients. In the end, we extracted the hazard ratios (HRs) and corresponding 95% confidence intervals (CIs) for overall survival (OS), disease-free survival (DFS)/recurrence-free survival (RFS), disease-specific survival (DSS) from each study. If a study conducts both univariate and multivariate analysis of variance, the results of the multivariate analysis will be used in further meta-analysis.

The quality of the included studies was evaluated using the Newcastle-Ottawa Quality Assessment Scale (NOS) [16]. Studies with scores equal to or higher than 6 points can be used for further meta-analysis. Two authors (Luyuan Ma and Rongyang Li) independently appraised the quality of each study, and all disagreements were resolved by consulting other researchers.

### Statistical analysis

The prognostic significance of TLS in patients with NSCLC was evaluated by calculating the aggregated HRs and 95% CIs, and the association between TLS and clinicopathological features in patients was evaluated by the aggregated odds ratio (ORs) and 95% CIs. In cases where studies displayed Kaplan-Meier curves but did not provide HRs or 95% CIs, we determined the HRs and 95% CIs by analyzing the survival curves with Engauge Digitizer V4.1 (Markmitch, Goteborg, Sweden) [17]. To reduce possible bias, a random effects model was used to calculate the overall effect size. The degree of heterogeneity was measured using the Cochrane Q test and I² statistics, where I² values exceeding 50% were deemed to indicate significant heterogeneity. Subgroup analysis was performed to identify the source of heterogeneity. Potential publication bias was assessed by Egger’s and Begg’s test. In order to confirm the stability of the combined results, we conducted a sensitivity analysis to assess how each study influenced the overall estimate by omitting individual studies in turn. A bilateral P value less than 0.05 was deemed statistically significant. All statistical analyses were executed by Stata software (version 15.1; Stata Corp., College Station, Texas, USA) and Review Manager software (RevMan version 5.3, the Nordic Cochrane Center, the Cochrane Collaboration, 2014).

## Results

### Literature search

**Fig. 1:**
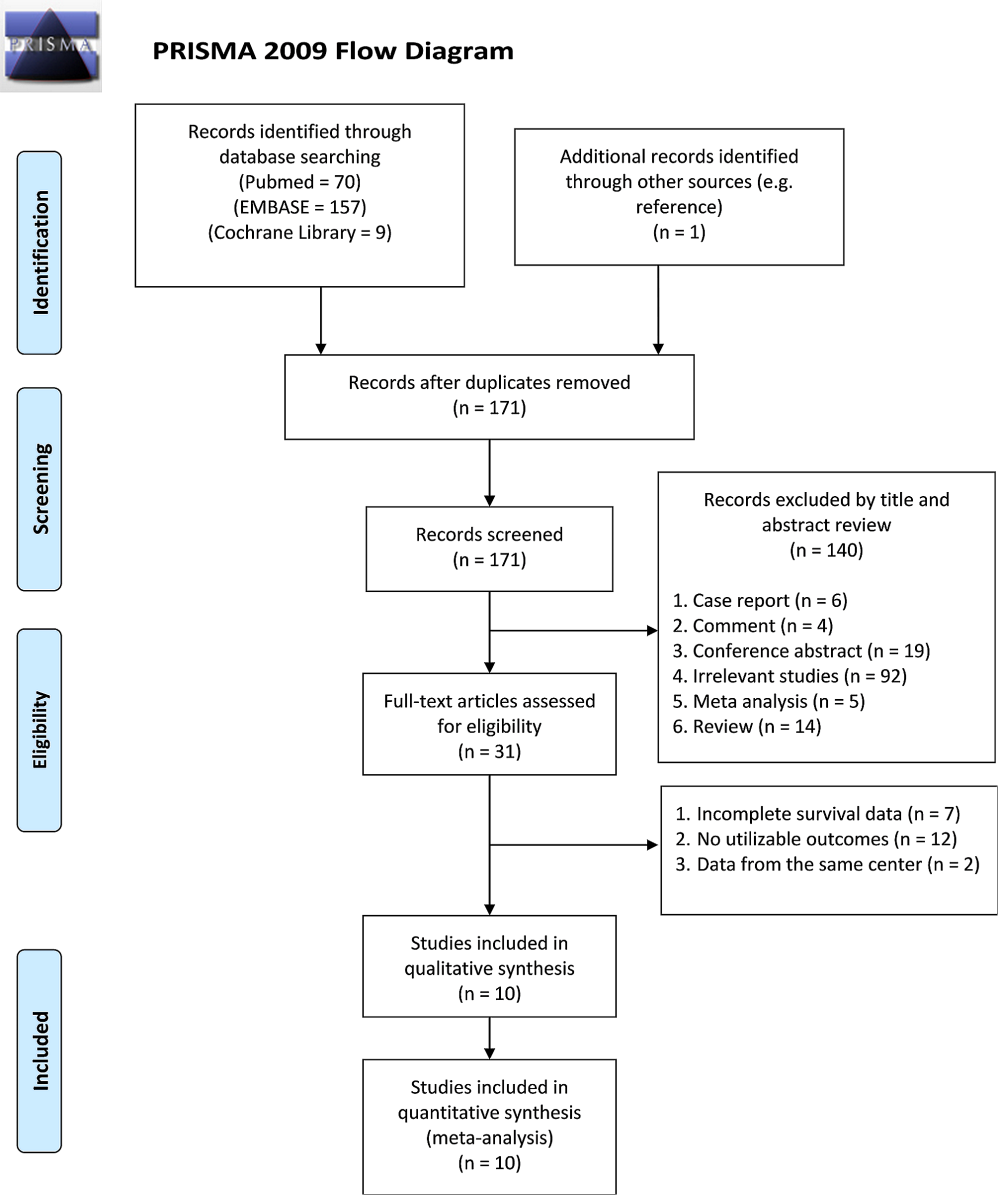
PRISMA flow diagram of literature search. PRISMA, Preferred Reporting Items for Systematic Reviews and Meta-Analyses

Through the literature search scheme, 237 documents with potential research value were retrieved, including 70 PubMed citations, 157 EMBASE citations, 9 Cochrane Library citations, and 1 relevant study yielded from the reference list. After eliminating duplicate publications, there were 171 studies left. By sifting through the titles and abstracts of each study, 31 studies remained. Finally, we carefully read the full text of the remaining articles, and 10 studies with 1,451 patients were included in our meta-analysis. A diagram illustrating the literature search process is shown in Fig. 1.

### Characteristics of the included studies

Table 1 describes the baseline characteristics and methodological assessments of each included study. There are ten retrospective studies published range from 2008 to 2023 from various regions of the globe. Four were published in China, two in Japan, two in America, one in Greece and one in Spain. The sample size of the study ranged from 59 to 490. It must be mentioned that all the eligible studies focused on intratumoral TLS, thus we mainly discuss the influence of intratumoral TLS on the prognosis of NSCLC patients. Of the included studies, patients in eight studies received surgical treatment only, and patients in three studies received neoadjuvant chemoimmunotherapy (NCIT) and surgical treatment. Notably, the study of Sun et al. examined both surgery-only and surgery with NCIT patients in relation to TLS, so we analyzed it as two studies [18]. In these ten studies, four evaluated the correlation between TLS and OS [19–22], seven evaluated the correlation between TLS and DFS/RFS [12, 13, 18, 19, 22–24], two evaluated the correlation between TLS and DSS [19, 25]. The studies that were included had NOS scores ranging from 7 to 9, suggesting that they are of high overall quality. Detailed quality assessments are presented in Supplementary Table 2.

**Table 1:** Baseline characteristics and methodological assessment of included studies

Author	Year	Country	Study design	Sample size	Stage	Treatment	TLS detection methods	TLS location	Cut-off criteria of TLS	Follow-up time (months)	Outcome
Alexandra et al.	2022	Greece	Retrospective	103	I-IV	Surgery	H-E staining	Global	Low/high	35 (3-102)	OS
Caroline et al.	2008	America	Retrospective	89	I-II	Surgery	IHC for DC	tumor tissue	Low/high	48	OS DSS DFS
Xu et al.	2023	China	Retrospective	117	I-IV	NCIT + Surgery	IHC	tumor tissue	Negative/positive	NR	DFS
Rakaee et al.	2021	America	Retrospective	490	I-III	Surgery	IHC for CD8/CK	tumor tissue	Negative/positive	84 (34–267)	DSS
Fukuhara et al.	2022	Japan	Retrospective	147	I-IV	Surgery	IHC for HEV	tumor tissue	Negative/positive	35	DFS
Tang et al.	2020	Spain	Retrospective	133	I-IV	Surgery	IHC	tumor tissue	Low/high	37.9 (20.0-65.4)	OS
Sun et al.	2022	China	Retrospective	121	I-IV	NCIT + Surgery/Surgery	IHC	tumor tissue	Negative/positive	24	DFS
Yang et al.	2020	China	Retrospective	59	I-IIIa	Surgery	IHC	tumor tissue	Low/high	36	DFS
Yutaro et al.	2022	Japan	Retrospective	112	Ib	Surgery	H-E staining	tumor tissue	Negative/positive	66.3	OS RFS
Liu et al.	2023	China	Retrospective	80	Ib-IIIb	NCIT + Surgery	H-E staining	tumor tissue	Low/high	17.5	DFS

TLS, tertiary lymphoid structure; NCIT, neoadjuvant chemoimmunotherapy; NR, not reported; H-E, heematoxylin-eeosin; IHC, Immunohistochemistry; OS, overall survival; DFS, disease-free survival; DSS, disease specific survival; RFS, recurrence-free survival

### Prognostic value of TLS in patients with NSCLC

**Fig. 2:**
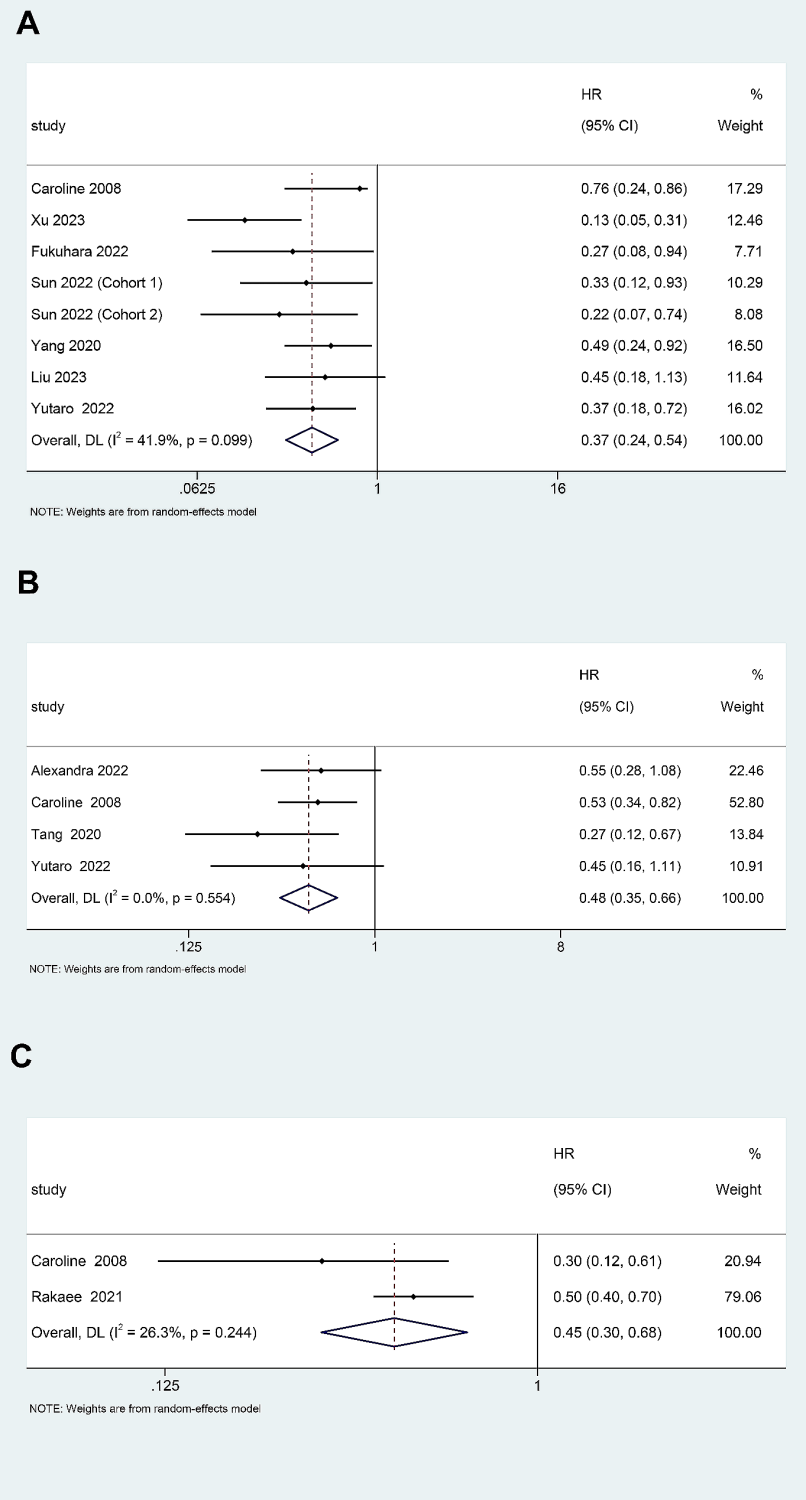
Forest plot of the correlation between TLS and (**A**) disease-free survival/recurrence-free survival, (**B**) overall survival, and (**C**) disease-specific survival in non-small cell lung cancer patients. TLS, tertiary lymphoid structures; HR, hazard ratio; CI, confidence interval

Eight studies involving 670 patients appraised the correlation between intratumoral TLS and DFS/RFS in NSCLC patients [12, 13, 18, 19, 22–24]. Pooled results revealed that high level of TLS is significantly associated with more favorable DFS/RFS (HR = 0.37, 95% CI: 0.24–0.54, *p* < 0.001) (Fig. 2A), with insignificant heterogeneity (I^2^ = 41.9%, *p* = 0.099). Subgroup analyses were conducted according to the treatment methods, TLS detection methods, and assessment of TLS cut-off values. The results showed that patients treated with neoadjuvant chemoimmunotherapy and surgery was correlated with better DFS/RFS, patients who used immunohistochemical (IHC) staining to detect TLS and those who used negative and positive TLS grouping had a better prognosis (Table 2, and Supplementary Fig. 1).

**Table 2:** Subgroup analyses of DFS/RFS in non-small cell lung cancer

Variable	No. of studies	No. of patients	Effects model	HR (95% CI)	*P*	Heterogeneity	
						I^2^, %	*P*
**DFS/RFS**							
All	8	670	Random	0.37 (0.24–0.54)	< 0.001	41.9	0.099
Detection methods							
H-E	4	345	Random	0.51 (0.36–0.73)	< 0.001	0.0	0.491
IHC	4	325	Random	0.21 (0.12–0.36)	< 0.001	0.0	0.547
Treatment							
Primary surgery	5	432	Random	0.48 (0.34–0.67)	< 0.001	0.0	0.431
NCIT + Surgery	3	238	Random	0.23 (0.11–0.50)	< 0.001	44.7	0.164
Cut-off criteria							
Low/high	3	213	Random	0.58(0.38–0.88)	0.010	0.0	0.539
Negative/positive	5	457	Random	0.26 (0.17–0.39)	< 0.001	0.0	0.447

RFS, recurrence-free survival; DFS, disease-free survival; H-E, hematoxylin-eosin staning; IHC, Immunohistochemistry; HR, hazard ratio; CI, confidence interval; NCIT, neoadjuvant chemoimmunotherapy

Four studies appraised the association between intratumoral TLS and OS in 422 patients [19–22]. The pooled analysis revealed that high TLS was associated with preferable OS (HR = 0.48, 95% CI: 0.35–0.66, *p* < 0.001). The heterogeneity of the studies was low (I^2^ = 0.0%, *p* = 0.554) (Fig. 2B). Only two studies have appraised the association between intratumoral TLS and DSS in NSCLC patients [19, 25]. The results indicate that high TLS is closely related to batter DSS (HR = 0.45, 95% CI: 0.30–0.68, *p* < 0.001), with low heterogeneity (I^2^ = 26.3%, *p* = 0.244) (Fig. 2C).

### Correlation between TLS and clinicopathological characteristics in NSCLC

**Fig. 3:**
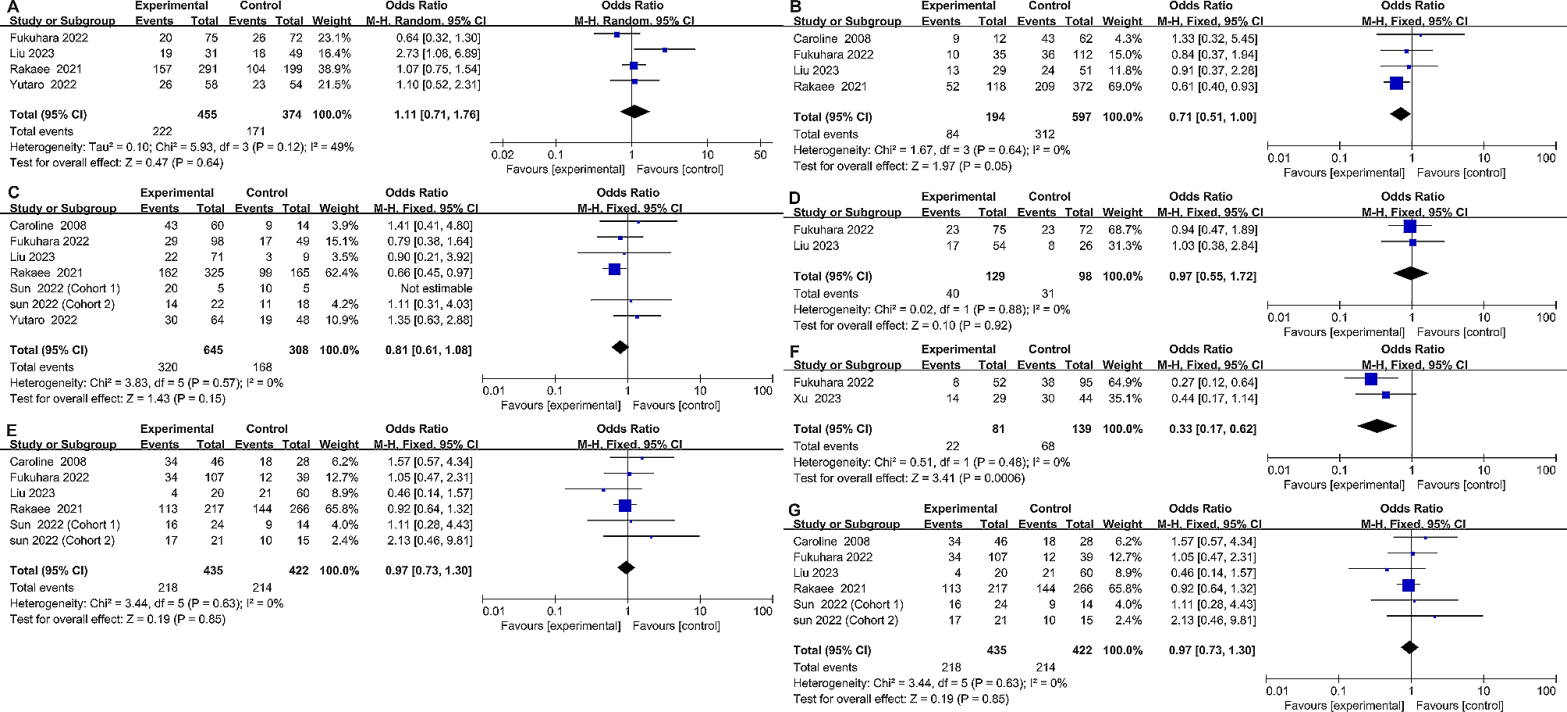
Forest plot of the correlation between TLS and clinicopathological characteristics in patients with NSCLC. (**A**) age; (**B**) TNM stage; (**C**) gender; (**D**) tumor size; (**E**) smoke; (**F**) NLR; (**G**) histological type. TLS, tertiary lymphoid structure; OR, odds ratio; CI, confidence interval; TNM, Tumor Node Metastasis; NLR, Neutrophil-lymphocyte ratio

The correlation analysis and evaluation results between TLS and various clinicopathological features are shown in Table 3. Overall, we examined the patients’ age (elder vs. young), gender (male vs. female), histological type (adenocarcinoma vs. squamous cell carcinoma), tumor size (large vs. small), smoking history (ever vs. never), TNM stage (II-IV vs. I), and NLR levels (high vs. low). After careful investigation, we determined that the relationship between TLS and TNM stage (OR = 0.71, 95% CI: 0.51-1.00, *p* < 0.05) and NLR level (OR = 0.33, 95% CI: 0.17–0.62, *p* < 0.001) was significant. TLS did not show any notable correlation with the patient’s age (OR = 1.11, 95% CI: 0.71–1.76, *p* = 0.64), gender (OR = 0.81, 95% CI: 0.61–1.08, *p* = 0.15), tumor classification (OR = 0.97, 95% CI: 0.73–1.30, *p* = 0.85), tumor size(OR = 0.97, 95% CI: 0.55–1.72, *p* = 0.92), or smoking status(OR = 1.01, 95% CI: 0.67–1.51, *p* = 0.97) (Fig. 3).

**Table 3:** Correlations of clinicopathological characteristics in patients with non-small cell lung cancer

Characteristics	No. of studies	No. of patients	Effects model	OR (95% CI)	*P*	Heterogeneity	
						I^2^, %	*P*
Age (elder vs. young)	4	829	Random	1.11 (0.71–1.76)	0.64	49	0.12
Sex (male vs. female)	7	938	Random	0.81 (0.61–1.08)	0.15	0.0	0.51
Histology (LUAD vs. LUSC)	6	857	Random	0.97 (0.73–1.30)	0.85	0.0	0.63
Smoke (ever vs. never)	6	903	Random	1.01 (0.67–1.51)	0.97	0.0	0.91
TNM stage (II-IV vs. I)	4	791	Random	0.71 (0.51-1.00)	< 0.05	0.0	0.64
Size (large vs. small)	2	227	Random	0.97 (0.55–1.72)	0.92	0.0	0.88
NLR (high vs. low)	2	264	Random	0.33 (0.17–0.62)	< 0.001	0.0	0.48

TNM, Tumor Node Metastasis; NLR, neutrophil-lymphocyte ratio; OR, odds ratio; CI, confidence interval; LUAD, Lung adenocarcinoma; LUSC, Lung squamous cell carcinoma

### Sensitive analysis and publication bias

We conducted a sensitivity analysis by excluding the studies one by one. The HRs calculated from the combined results of the remaining studies in each analysis did not go beyond the expected range, as illustrated in Supplementary Fig. 2 and Supplementary Fig. 3. There is no significant difference between the revised overall estimate and the primary combined estimate, indicating that the meta-analysis is reliable. Begg’s and Egger’s tests are employed to identify any potential publication bias. The meta-analysis did not show any clear publication bias on TLS with respect to OS (Egger’s *p* = 0.369, Begg’s *p* = 0.308) and DFS/RFS (Egger’s *p* = 0.117, Begg’s *p* = 0.117).

## Discussion

In the past few years, as researchers have delved deeper into the tumor microenvironment (TME) and the workings of tumor immunotherapy, TLS has emerged as a significant biological structure that hinders tumor growth by stimulating the activation of immune cells near the tumor [4, 26, 27]. Numerous research studies have investigated the significance of TLS in treating individuals with cancers, and the majority indicating that elevated TLS levels are a crucial indicator of a positive prognosis for various solid tumors [28, 29]. However, the prognostic value of TLS in NSCLC remains controversial. This meta-analysis integrated prognostic data and clinical characteristics of 1,451 NSCLC patients from 10 studies and conducted subgroup analysis. Following a thorough quantitative analysis of prognostic data, we determined that elevated levels of TLS were strongly associated with improved OS, DSS, and DFS/RFS. Additionally, high TLS levels were found to be closely linked to the tumor TNM stage and NLR. This meta-analysis represents the most up-to-date and extensive investigation regarding the correlation between TLS and prognosis, as well as relevant clinicopathological characteristics in individuals diagnosed with NSCLC.

Although only three studies in this meta-analysis focused on the relationship between TLS and patient outcomes in those who underwent immunotherapy before surgery, our findings indicated that individuals with increased TLS levels who received immunotherapy before surgery had a more favorable prognosis compared to those who underwent surgery alone [30]. TLS is an essential component of the tumor immune microenvironment (TIME) and includes T cells, B cells, fibroblast reticular cells (FRC) networks, high endothelial venules (HEV), and follicular dendritic cells (FDC) [4, 31, 32]. Within the TME, TLS serves as a site where immune cells can proliferate and interact. This area is primarily made up of an internal region of CD20+ B cells and a surrounding region of CD3+ T cells [33, 34]. Additionally, there is a significant presence of dendritic cells (DC) surrounding the immune cells, all of which congregate in this space to collectively suppress tumor growth. In this cluster of immune cells, DC displays the surface antigen of nearby tumor tissue to T cells via TLS [35, 36]. The activated T cells then produce memory helper T cells and effector memory cytotoxic cells to aid in the destruction of tumor cells through phagocytosis [37, 38]. Furthermore, this cluster supports the activation and growth of B cells, facilitating the development, activation, and growth of memory B cells and plasma cells [39, 40]. These immune cells further contribute to the body’s ability to eliminate tumor cells by generating antibodies. Tumor infiltrating lymphocytes (TILs) are lymphocytes isolated from tumor tissue. It plays a key role in the host antigen-specific tumor immune response [41], and the adoptive immunotherapy approach mediated by TILs has achieved good efficacy in a variety of solid tumors [42, 43]. It has been reported that TLS and TILs play similar roles in the anti-tumor process. However, the study we included found that although there was a certain relationship between the density of TLS and TILs, the joint increase of the two did not have a synergistic effect on the prognosis of the tumor, but were independent of each other [19, 20]. Moreover, Cottrel et al. found that the presence of TLS within the tumor area was consistently associated with cellular apoptosis in patients exhibiting a favorable response to preoperative immune checkpoint inhibitor therapy. Conversely, nonspecific collection of TILs unrelated to the treatment response was also observed [44]. This implies that TLS, rather than TILs, could serve as a more reliable indicator of the therapeutic efficacy for NSCLC patients. Hence, elevated levels of intratumoral TLS could serve as a significant prognostic indicator for NSCLC patients. This further validates the connection between TLS and the immune mechanisms within the tumor microenvironment, highlighting an important area for future investigation.

Currently, there is a lack of consistent criteria globally for choosing TLS detection techniques and determining threshold values, which poses a significant challenge to utilizing TLS as a key prognostic indicator [45]. To identify the most effective approach for assessing TLS, we carried out a subgroup analysis of the studies included. In this meta-analysis, there are variations in how TLS is detected and the cutoff methods used across different studies. According to our analysis results, the use of IHC staining to detect TLS and its grouping by negative or positive results both suggest that patients have a better prognosis. This is probably because these two techniques more accurately reflect the levels of TLS in the patient’s body. Therefore, IHC, along with categorizing TLS as negative or positive, could be potentially used together as a standard method for identifying and assessing TLS. However, due to the limited sample size in the studies included, additional research is required to gather more evidence before it can be established as a universal standard for evaluating.

Nevertheless, this meta-analysis has certain constraints. Primarily, most of the included studies were retrospective cohort studies conducted at a single center, potentially leading to biases such as cohort selection bias that could impact the reliability of the findings. Moreover, variations in the methods used to establish TLS cutoff values among the included studies could result in selection bias and diverse outcomes. Furthermore, certain studies lacked precise prognostic details, prompting us to utilize Engauge Digitizer software to estimate the survival statistics of select studies by analyzing the survival curve. This method may yield results that differ from the original data. Moreover, there are only a few studies that can be used for subgroup analysis, particularly within the immunotherapy subgroup. Merely three immunotherapy studies were incorporated, and the sample size was relatively small, suggesting potential inaccuracies in our assessment of immunotherapy [12, 13, 18]. Finally, our meta-analysis focused solely on the presence of TLS within the tumor itself, rather than its presence outside the tumor, which may not fully represent its impact on tumor prognosis. Given these constraints, it is essential to conduct numerous multi-center prospective studies to validate our findings before implementing them in clinical settings.

## Conclusion

TLS plays a crucial role in the treatment of NSCLC. Elevated TLS levels are strongly related to positive survival outcomes such as OS, DSS, and DFS/RFS in NSCLC. Additionally, TLS expression levels are closely associated with certain clinicopathological factors of NSCLC patients. Therefore, TLS has the potential to serve as a biomarker for predicting the prognosis of NSCLC patients and may influence clinical treatment decisions. Nevertheless, further prospective studies are necessary to validate the prognostic significance of TLS in NSCLC patients before its clinical application.

### Electronic supplementary material

Below is the link to the electronic supplementary material.

Supplementary Material 1

Supplementary Material 2

**Supplementary Material 3: Supplementary Figure 1.** Subgroup analysis of the correlation between TLS in non-small cell lung cancer patients based on (A) the type of tumor treatment, and (B) assessment of TLS cutoff values, and (C) TLS detection methods. TLS, tertiary lymphoid structures; NCIT, neoadjuvant chemoimmunotherapy; IHC, immunohistochemical; H-E, Hematoxylin and eosin; OR, odds ratio; CI, confidence interval

**Supplementary Material 4: Supplementary Figure 2.** Sensitivity analysis of (A) disease-free survival/recurrence-free survival, (B) overall survival,(C) disease-specific survival

**Supplementary Material 5: Supplementary Figure 3.** Sensitivity analysis of (A) age; (B) TNM stage; (C) gender; (D) tumor size; (E) smoke; (F) NLR; (G) histological type. TNM, Tumor Node Metastasis; NLR, Neutrophil-lymphocyte ratio

## Data Availability

All data are generated from public data, which has been shown in the article. The data that support the findings of this study are available on request from the corresponding author.

## Abbreviations

NSCLC: Non-small cell lung cancer
TLS: Tertiary lymphoid structure
HRs: Hazard ratios
ORs: Odds ratios
CIs: Confidence intervals
OS: Overall survival
DFS: Disease-free survival
RFS: Recurrence-free survival
DSS: Disease-specific survival
TNM: Tumor Node Metastasis
NLR: Neutrophil-lymphocyte ratio
SLO: Secondary lymphoid organ
MeSH: Medical subject terms
NOS: Newcastle-Ottawa Quality Assessment Scale
NCIT: Neoadjuvant chemoimmunotherapy
IHC: Immunohistochemical
H-E: Hematoxylin and eosin
TME: Tumor microenvironment
TIME: Tumor immune microenvironment
FRC: Fibroblast reticular cells
HEV: High endothelial venules
FDC: Follicular dendritic cells
DC: Dendritic cells
TILs: Tumor infiltrating lymthocytes
