# Supplementary material for: Prognostic and clinicopathological significance of tertiary lymphoid structure in non-small cell lung cancer: a systematic review and meta-analysis
Source: BMC Cancer. 2024 Jul 8;24:815. doi: 10.1186/s12885-024-12587-x (PMC11229181; doi:10.1186/s12885-024-12587-x)
Supplement: Supplementary file 1 — Supplementary Material 1 [file 12885_2024_12587_MOESM1_ESM.docx]

**Supplementary Table 1. Detailed search strategies for three databases.**

| **Database** | **Search strategies** |
| --- | --- |
| Pubmed | ((((((((((((Tertiary lymphoid structure[Title/Abstract]) OR (Tertiary lymphoid structures[Title/Abstract])) OR (Tertiary lymphoid organ[Title/Abstract])) OR (Tertiary lymphoid tissue[Title/Abstract])) OR (Ectopic lymphoid like structure[Title/Abstract])) OR (Ectopic lymphoid like structures[Title/Abstract])) OR (Ectopic lymphoid organ[Title/Abstract])) OR (Ectopic lymphoid follicle[Title/Abstract])) OR (Ectopic lymphoid formations[Title/Abstract])) OR (TLS[Title/Abstract])) OR (TLSs[Title/Abstract])) AND ((((((((((Pulmonary Neoplasms[Title/Abstract]) OR (Pulmonary Neoplasm[Title/Abstract])) OR (Lung Neoplasm[Title/Abstract])) OR (Lung Neoplasms[Title/Abstract])) OR (Lung Cancer[Title/Abstract])) OR (Lung Cancers[Title/Abstract])) OR (Pulmonary Cancer[Title/Abstract])) OR (Pulmonary Cancers[Title/Abstract])) OR (Cancer of the Lung[Title/Abstract])) OR (Cancer of Lung[Title/Abstract]))) AND ((((Prognosis[Title/Abstract]) OR (Prognostic[Title/Abstract])) OR (Survival[Title/Abstract])) OR (Outcome[Title/Abstract])) |
| Embase | ('tertiary lymphoid structure':ab,ti OR 'tertiary lymphoid structures':ab,ti OR 'tertiary lymphoid organ':ab,ti OR 'tertiary lymphoid tissue':ab,ti OR 'ectopic lymphoid like structure':ab,ti OR 'ectopic lymphoid like structures':ab,ti OR 'ectopic lymphoid organ':ab,ti OR 'ectopic lymphoid follicle':ab,ti OR 'ectopic lymphoid formations':ab,ti OR 'tls':ab,ti OR 'tlss':ab,ti) AND ('pulmonary neoplasms':ab,ti OR 'pulmonary neoplasm':ab,ti OR 'lung neoplasm':ab,ti OR 'lung neoplasms':ab,ti OR 'lung cancer':ab,ti OR 'lung cancers':ab,ti OR 'pulmonary cancer':ab,ti OR 'pulmonary cancers':ab,ti OR 'cancer of the lung':ab,ti OR 'cancer of lung':ab,ti) AND (prognosis:ab,ti OR prognostic:ab,ti OR survival:ab,ti OR outcome:ab,ti) |
| Cochrane | (("Tertiary lymphoid structure"):ti,ab,kw OR ("Tertiary lymphoid structures"):ti,ab,kw OR ("Tertiary lymphoid organ"):ti,ab,kw OR ("Tertiary lymphoid tissue"):ti,ab,kw OR ("Ectopic lymphoid like structure"):ti,ab,kw OR ("Ectopic lymphoid like structures"):ti,ab,kw OR ("Ectopic lymphoid organ"):ti,ab,kw OR ("Ectopic lymphoid follicle"):ti,ab,kw OR ("Ectopic lymphoid formations"):ti,ab,kw) AND (("Pulmonary Neoplasms"):ti,ab,kw OR ("Pulmonary Neoplasm"):ti,ab,kw OR ("Lung Neoplasm"):ti,ab,kw OR ("Lung Neoplasms"):ti,ab,kw OR ("Lung Cancer"):ti,ab,kw OR ("Lung Cancers"):ti,ab,kw OR ("Pulmonary Cancer"):ti,ab,kw OR ("Pulmonary Cancers"):ti,ab,kw OR ("Cancer of the Lung"):ti,ab,kw OR ("Cancer of Lung"):ti,ab,kw) AND ((Prognosis):ti,ab,kw OR (Prognostic):ti,ab,kw OR (Survival):ti,ab,kw OR (Outcome):ti,ab,kw) |
