# Supplementary material for: Prognostic and clinicopathological significance of tertiary lymphoid structure in non-small cell lung cancer: a systematic review and meta-analysis
Source: BMC Cancer. 2024 Jul 8;24:815. doi: 10.1186/s12885-024-12587-x (PMC11229181; doi:10.1186/s12885-024-12587-x)
Supplement: Supplementary file 2 — Supplementary Material 2 [file 12885_2024_12587_MOESM2_ESM.docx]

| **Items of NOS** | **Included Studies** | | | | | | | | | | |
| --- | --- | --- | --- | --- | --- | --- | --- | --- | --- | --- | --- |
|  | Alexandra 2022 | Caroline2008 | Xu 2023 | Rakaee 2021 | Tang 2020 | Fukuhara2022 | Sun 2022 | | Yang  2020 | Yutaro 2022 | Liu  2023 |
| **Selection** |  |  |  |  |  |  |  |  | |  |  |
| Representativeness of the exposed cohort | **★** | **★** | **★** | **★** | **★** | **★** | **★** | **★** | | **★** | **★** |
| Selection of the non-exposed cohort | **★** | **★** | **★** | **★** | **★** | **★** | **★** | **★** | | **★** | **★** |
| Ascertainment of exposure | **★** | **★** | **★** | **★** | **★** | **★** | **★** | **★** | | **★** | **★** |
| Demonstration that outcome of interest was not present at start of study | **★** | **★** | **★** | **★** | **★** | **★** | **★** | **★** | | **★** | **★** |
| **Comparability** |  |  |  |  |  |  |  |  | |  |  |
| Comparability of cohorts on basis of the design or analysis |  | **★★** | **★** | **★★** | **★** | **★★** | **★★** |  | | **★** | **★★** |
| **Outcome** |  |  |  |  |  |  |  |  | |  |  |
| Assessment of outcome | **★** | **★** | **★** | **★** | **★** | **★** | **★** | **★** | | **★** | **★** |
| Follow-up long enough for outcomes to occur | **★** | **★** | **★** | **★** | **★** | **★** | **★** | **★** | | **★** | **★** |
| Adequacy of follow up of cohorts | **★** | **★** | **★** | **★** | **★** | **★** | **★** | **★** | | **★** | **★** |
| **Total** | **7** | **9** | **8** | **9** | **8** | **9** | **9** | **7** | | **8** | **9** |

**Table S2.** Detailed quality assessment of cohort studies.

A study can be awarded a maximum of one star for each numbered item within the Selection and Outcome categories.

A maximum of two stars can be given for Comparability. Study rates ≥6 is eligible for further analysis. NOS, Newcastle-Ottawa Scale.
