# Supplementary figures and images for: Prognostic and clinicopathological significance of tertiary lymphoid structure in non-small cell lung cancer: a systematic review and meta-analysis
Source: BMC Cancer. 2024 Jul 8;24:815. doi: 10.1186/s12885-024-12587-x (PMC11229181; doi:10.1186/s12885-024-12587-x)

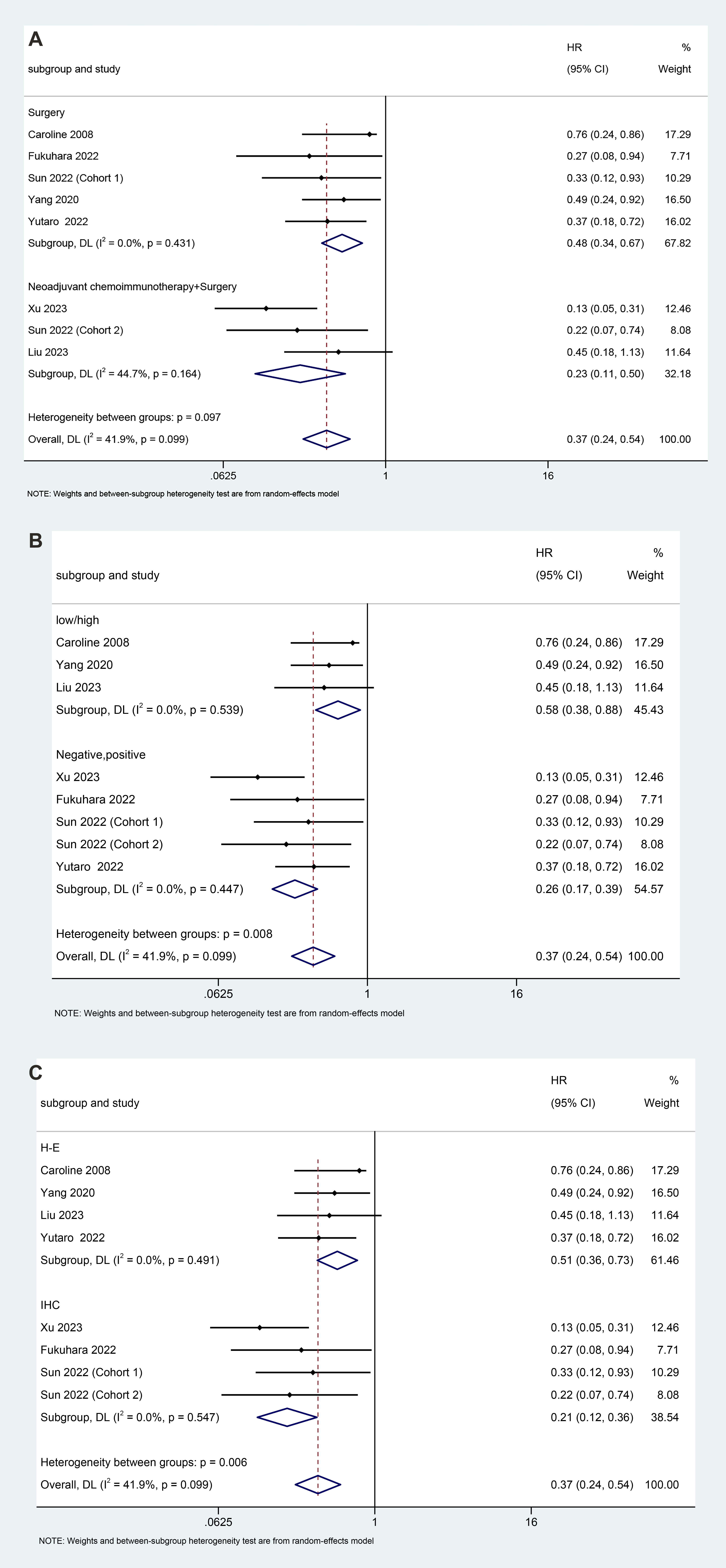

Supplement: Supplementary file 3 — Supplementary Material 3: Supplementary Figure 1. Subgroup analysis of the correlation between TLS in non-small cell lung cancer patients based on (A) the type of tumor treatment, and (B) assessment of TLS cutoff values, and (C) TLS detection methods. TLS, tertiary lymphoid structures; NCIT, neoadjuvant chemoimmunotherapy; IHC, immunohistochemical; H-E, Hematoxylin and eosin; OR, odds ratio; CI, confidence interval [file 12885_2024_12587_MOESM3_ESM.png]

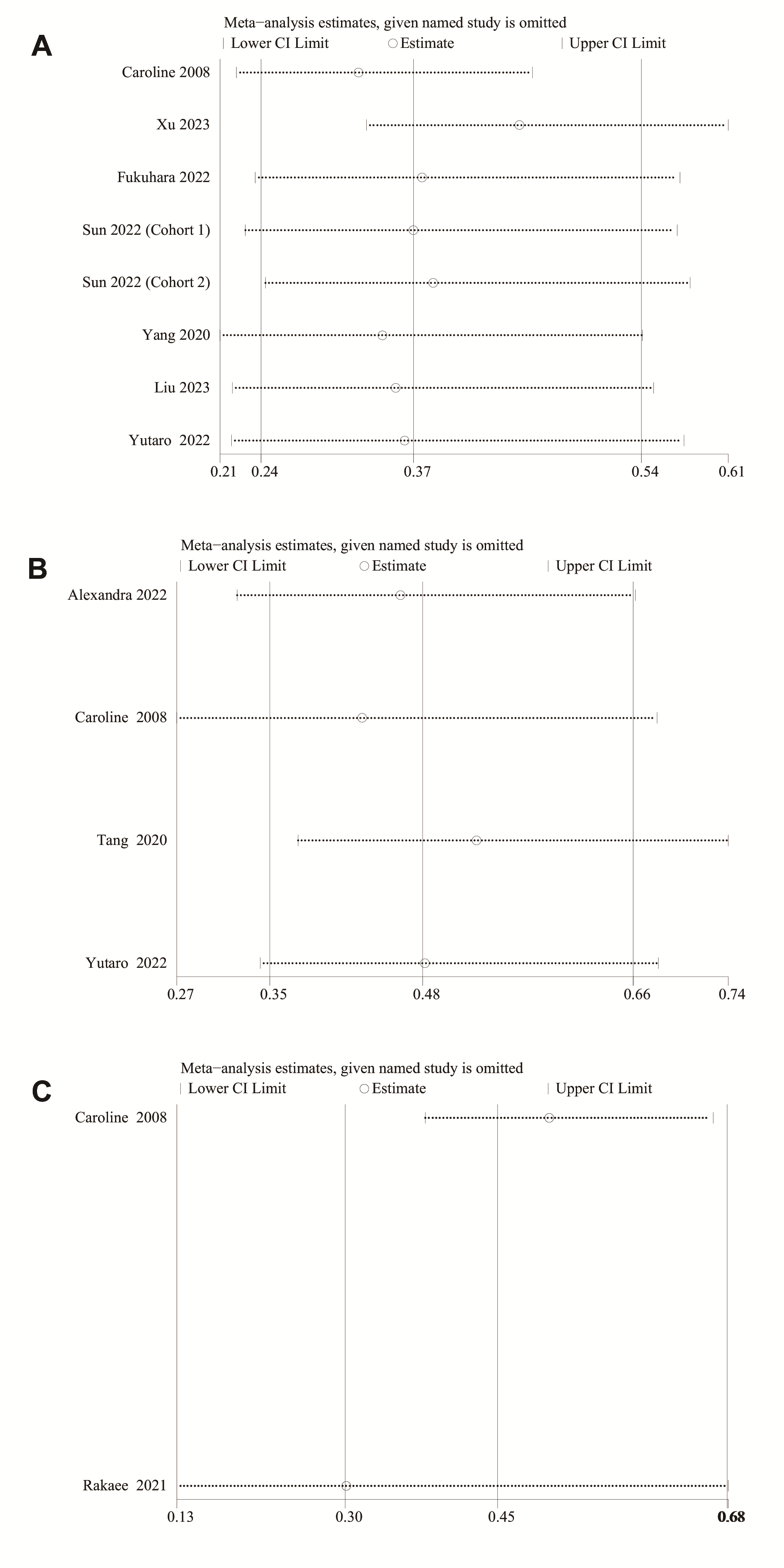

Supplement: Supplementary file 4 — Supplementary Material 4: Supplementary Figure 2. Sensitivity analysis of (A) disease-free survival/recurrence-free survival, (B) overall survival,(C) disease-specific survival [file 12885_2024_12587_MOESM4_ESM.png]

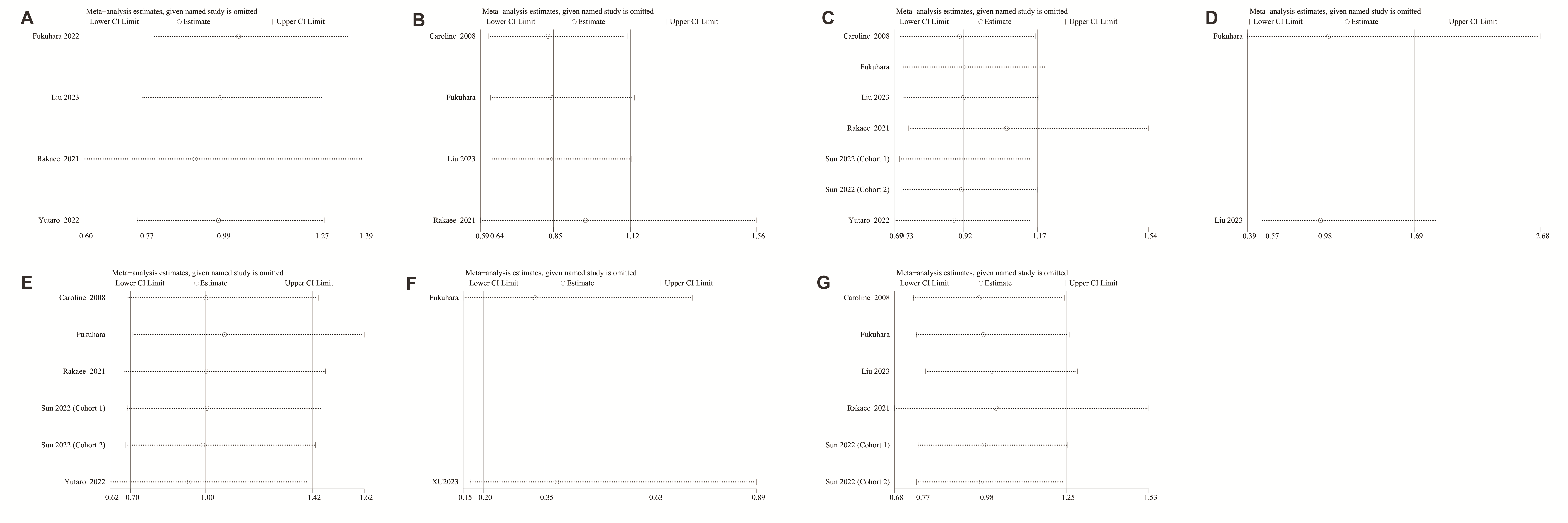

Supplement: Supplementary file 5 — Supplementary Material 5: Supplementary Figure 3. Sensitivity analysis of (A) age; (B) TNM stage; (C) gender; (D) tumor size; (E) smoke; (F) NLR; (G) histological type. TNM, Tumor Node Metastasis; NLR, Neutrophil-lymphocyte ratio [file 12885_2024_12587_MOESM5_ESM.png]
